# P2X7 Receptor Expression and Signaling on Dendritic Cells and CD4^+^ T Cells is Not Required but Can Enhance Th17 Differentiation

**DOI:** 10.3389/fcell.2022.687659

**Published:** 2022-03-08

**Authors:** Yin Yang, Meaghan E. Story, Xingxing Hao, Tina L. Sumpter, Alicia R. Mathers

**Affiliations:** ^1^ Department of Dermatology, University of Pittsburgh School of Medicine, Pittsburgh, PA, United States; ^2^ Department of Immunology, University of Pittsburgh School of Medicine, Pittsburgh, PA, United States

**Keywords:** P2X7R, IL-17, Th17, Th1, dendritic cells, purinergic signaling, CD4 T cell

## Abstract

The purinergic receptor P2X7 (P2X7R) is important in inflammasome activation and generally considered to favor proinflammatory immune responses. However, there is still a limited understanding of the role of P2X7R signaling in Th cell differentiation, particularly, Th17 differentiation. Herein, the impact of P2X7R signaling on primary Th17 and Th1 cell responses was examined when P2X7R was expressed specifically on dendritic cells (DCs) and CD4^+^ T cells. Surprisingly, global genetic ablation and pharmacological inhibition of the P2X7R did not affect the generation of Th17 and Th1 development in response to immunization with Complete Freund’s Adjuvant and the model antigens, keyhole limpet hemocyanin or OVA. However, in-depth *in vitro* and *in vivo* investigations revealed differences in the balance of Th1/Th17 differentiation when P2X7R blockade was restricted to either DCs or CD4^+^ T cells. In this regard, *in vitro* DCs treated with a P2X7R agonist released more IL-6 and IL-1β and induced a more robust Th17 response in mixed leukocyte reactions when compared to controls. To test the hypothesis that P2X7R signaling specifically in DCs enhances Th17 responses *in vivo,* DC-specific P2X7R deficient chimeras were immunized with CFA and OVA. In this model, the P2X7R expression on DCs decreased the Th1 response without impacting Th17 responses. Following an assessment of CD4^+^ T cell P2X7R signaling, it was determined that *in vitro* P2X7R sufficient T cells develop an increased Th17 and suppressed Th1 differentiation profile. *In vivo*, P2X7R expression on CD4^+^ T cells had no effect on Th17 differentiation but likewise significantly suppressed the Th1 response, thereby skewing the immune balance. Interestingly, it appears that WT OT-II Th1 cells are more sensitive to P2X7R-induced cell death as evidence by a decrease in cell number and an increase in T cell death. Overall, these studies indicate that *in vitro* P2X7R signaling does enhances Th17 responses, which suggests that compensatory Th17 differentiation mechanisms are utilized *in vivo* in the absence of P2X7R signaling.

## Introduction

Adenosine triphosphate (ATP) is recognized as an intercellular mediator that orchestrates immune responses, in addition to its well-known role as energy currency and an intracellular second messenger. In this regard, ATP, an alarmin released from stressed, wounded, or necrotic cells, is considered a damage-associated molecular pattern (DAMP) that acts as a danger-signal initiating inflammation, including innate and adaptive immunity. Among the purinergic ATP receptors, P2X7R is widely expressed in mouse and human tissues and is physiologically necessary for triggering inflammation. Mechanistically, when P2X7R is gated with a low ATP concentration it allows Ca^2+^ and Na^+^ influx and K^+^ efflux, which supports cell growth and activation. However, a high concentration of ATP and prolonged stimulation will induce P2X7R to form a large nonselective membrane pore, which is linked to cell death and is dependent on the C-terminus of P2X7R (Cheewatrakoolpong et al., 2005; Di Virgilio et al., 2017). In mice, the cell death-inducing function can also be triggered by NAD^+^ through ADP-ribosylation of P2X7R by ADP-ribosyltransferase two on the plasma membrane (Seman et al., 2003; Rissiek et al., 2015).

A prototypical function of P2X7R signaling is the activation of the NLRP3 inflammasome in macrophages and monocytes, which leads to Caspase-1 activation and the processing and secretion of mature IL-1β (Di Virgilio et al., 2017). IL-1β is a proinflammatory cytokine involved in both innate and adaptive immune responses and inflammatory diseases (Mantovani et al., 2019). For instance, P2X7R has been implicated in many inflammatory diseases, including multiple sclerosis, colitis, rheumatoid arthritis, psoriasis, and glomerulonephritis, diseases which Th17 cells have been shown to play a dominant role in the immunopathogenesis (Weaver et al., 2013; Cao et al., 2019). In this regard, P2X7R contributes to Th17 responses in murine colitis, murine arthritis, murine and human psoriatic skin inflammation, allograft rejection, and human adipose tissue inflammation (Atarashi et al., 2008; Killeen et al., 2013; Fan et al., 2016; Pandolfi et al., 2016; Diaz-Perez et al., 2018; Fan et al., 2021). Mechanistically, studies suggest that P2X7R can regulate the function and development of many T cell subsets, including Th1, Th17, Treg, and Tfh cells (Rissiek et al., 2015; Di Virgilio et al., 2017). Likewise, Borges da Silva et al. demonstrate that P2X7R also promotes the maintenance of memory CD8^+^ T cells (Borges da Silva et al., 2018). However, our understanding of P2X7R’s effect on Th17 differentiation is incomplete. In this regard, reports also demonstrate that P2rx7^−/−^ mice develop more severe inflammation in the intestine during *Citrobacter rodentium* (Th17 and Th1) or *Toxoplasma gondii* (Th1) infection, and in the central nervous system in a model of experimental autoimmune encephalomyelitis (Th17) (Chen and Brosnan, 2006; Miller et al., 2015; Hashimoto-Hill et al., 2017). Factors contributing to this discrepancy may be a dichotomy that has been observed between P2rx7^−/−^ mice and pharmacological P2X7R inhibitors (De Marchi et al., 2019) and cell intrinsic functions attributed to P2X7R signaling.

The activation of CD4^+^ T cells requires the recognition of antigenic peptides in the context of MHC class II molecules and coordinated engagement of co-stimulatory molecules. Further, CD4^+^ T cell differentiation is directed by specific cytokine profiles secreted by dendritic cells (DCs) during Ag presentation (Banchereau and Steinman, 1998). Thus, to further our understanding of the role and impact of direct P2X7R signaling on T cell differentiation, we utilized both genetic and pharmacological techniques to separately examine the respective contributions of P2X7R expression on DCs and CD4^+^ T cells to the differentiation of Th17 cells using classical *in vitro* and *in vivo* adaptive immune models. We demonstrate that *in vitro* P2X7R expression on DCs and T cells can promote Th17 development, whereas in the context of an *in vivo* setting classical DC (cDC)-specific ablation of P2X7R did not impair Th17 development but did impact the Th1 response. Moreover, *in vivo* P2X7R expression on CD4^+^ T cells promoted cell death and therefore resulted in a comparable total number of Th17 cells and in both WT and P2rx7^−/−^ mice. Thus, these results indicate that P2X7R expression on either DCs or CD4^+^ T cells can enhance Th17 differentiation but that in a model system, compensatory pathways exist that maintain the Th17 response in the absence of P2X7R signaling and shift the Th1/Th17 balance.

## Materials and Methods

### Mice

B6.129P2-P2rx7^tm1Gab^/J (P2rx7^−/−^), C57BL/6J (CD45.2), B6. SJL (CD45.1), BALB/cJ, OT-II, and zDC-DTR mice were purchased from The Jackson Laboratory. P2rx7^−/−^ OT-II mice were generated by crossing P2rx7^−/−^ with OT-II mice. CD45.1 OT-II mice were obtained by breeding B6. SJL with OT-II mice. Mice were maintained in specific pathogen-free conditions and all experiments were performed in accordance with the University of Pittsburgh Institutional Animal Care and Use Committee and NIH’s Guide for Care and Use of Laboratory Animals.

### Antibodies and Flow Cytometry

The following antibodies were purchased from BD Biosciences (San Jose, CA): anti-CD16/CD32 (2.4G2), BUV395 anti-CD3e (145-2C11), BUV737 anti-CD45.1 (A20), PE anti-IFNγ(XMG1.2), Alexa Fluor 647 anti-IL-17A (TC11-18H10). The following antibodies were purchased from Biolegend (San Diego, CA): Alexa Fluor 488 or PerCP/Cy5.5 anti-CD4 (GK1.5), Brilliant Violet 605 anti-CD45.2 (104). Viability Dye eFluor 450 was purchased from eBioscience (San Diego, CA). For intracellular staining, surface-stained cells were fixed and permeabilized with BD Cytofix/Cytoperm kit following manufacturer’s instructions and stained intracellularly with designated cytokine antibodies. For indicated experiments, cell death was quantified as cells positive for Viability Dye. To determine absolute cell numbers, cells were collected from each sample and mixed with 100 µL of AccuCheck Counting Beads (Life technologies, Carlsbad, CA). Absolute cell number was calculated for each sample based on the percentage and number of beads and sample volume as per manufacturer’s instructions. Cells were analyzed using an LSR II flow cytometer or LSR Fortessa and FlowJo software was utilized for analysis (BD Biosciences).

### Generation of Bone Marrow Derived DCs (BMDCs)

Mouse bone marrow was isolated from femurs and tibias. RBCs were lysed and T and B lymphocytes were removed by complement depletion using a cocktail of monoclonal antibodies (anti–CD3, anti-B220; BD biosciences) and rabbit complement (Cedarlane, Burlington, Canada). Cells were cultured at 1.0 × 10^6^ cells/ml in complete RPMI-1640 supplemented with 20 ng/ml GM-CSF and 5 ng/ml IL-4 (PeproTech, Cranbury, NJ) for 7 days (replaced 70% of medium on Day 2 and Day 5). Loosely adherent BMDCs were collected and further purified with CD11c^+^ magnetic beads (Miltenyi Biotec, Bergisch Gladbach, Germany) to obtain a purity of >90%. BMDCs were stimulated with 100, 300, 500 µM of BzATP, P2X7R agonist (SigmaAldrich, St Louis, MO) for 3 h or 6h, as indicated, and supernatants were harvested for analysis of IL-6 and IL-1β by ELISA, performed according to manufacturer’s instructions (Biolegend). For some experiments, BMDCs were pre-stimulated with 300 µM BzATP for 5 h and thoroughly washed before coculture. BMDCs were also utilized in MLRs and *in vitro* T cell differentiation assays.

### Mixed Leukocyte Reaction (MLR)

BMDCs (1.0 × 10^4^) derived from wild-type C57BL/6J or P2rx7^−/−^ mice were cocultured with 1.0 × 10^5^ naive CD4^+^ T cells magnetically sorted from the spleen of BALB/cJ mice in 200 ul of complete RPMI-1640 in round-bottom 96-well plates. Supernatants were harvested and IL-17A was measured by ELISA, according to manufacturer’s instructions (Biolegend).

### Antigen Specific *in vivo* Immunizations

C57Bl/6J and P2rx7^−/−^ mice were immunized subcutaneously at the tail base with 20 µg OVA_323-339_ peptide (Anaspec, Fremont, CA) emulsified in CFA (BD Biosciences) or 20 µg KLH in CFA in indicated experiments. Some groups were treated with the selective P2X7R antagonist, A740003 (50 μg/kg diluted in PBS/0.55% DMSO; Tocris, Minneapolis, MN) or the vehicle control, i. p every other day for 7 days (De Marchi et al., 2019). On day 7, inguinal lymph nodes were collected and processed into single cell suspensions. For determination of IL-17A^+^ and IFNγ^+^ CD45.1^+^ OT-II CD4^+^ T cell percentages, cells were restimulated with 50 ng/ml PMA and 500 ng/ml ionomycin in the presence of GolgiPlug for 4 h then stained for flow cytometric analysis. For detection of cytokines in supernatants, cells were restimulated with cognate antigens (OVA, 10 μg/ml and KLH 50 μg/ml) for 3 days then supernatants were collected and IL-17A and IFNγ levels were assessed by ELISA, performed according to manufacturer’s instructions (Biolegend).

### Adoptive T Cell Transfer Studies

Naive CD45.1^+^ OT-II CD4^+^ T cells were isolated by magnetic sorting utilizing the mouse naive CD4^+^ T cell isolation kit and performed according to manufacturer’s instructions (Miltenyi Biotec, Germany). Isolated CD4^+^ T cells were transferred intravenously into congenic mice (1.0 × 10^5^ cells/mouse). The following day recipient mice were immunized as described in the above Immunizations section.

### Mixed Bone Marrow Chimera Studies

Recipient C57BL/6J mice were irradiated with two doses of 550 rad, 3 h apart. Mice were fed with Uniprim diet (Envigo, Indianapolis, IN) for 2 weeks to prevent infections. The day following irradiation, bone marrow was isolated from donor mice and RBCs and T cells were depleted. Cell density was similarly adjusted from each strain, between 2.0 × 10^7^ to 4.0 × 10^7^ cells/ml. Bone marrow cells from WT C57Bl/6J or P2X7R^−/-^ and zDC-DTR mice were mixed 1:1 and 4.0 × 10^6^ to 8.0 × 10^6^ cells were injected intravenously per recipient mouse. Between 8 and 12 weeks following reconstitution, naïve CD45.1^+^ OT-II CD4^+^ T cells were injected i. v. into each chimera. The day after T cell transfer, chimeras were injected i. p. with either PBS or 1.25 µg Diphtheria Toxin (Sigma), to deplete cDCs. To maintain DT-induced DTR^+^ cell depletion, DT (1.25 µg/mouse) was injected every other day for the duration of experiments. Chimeras were immunized with 100 µg OVA_323-339_ peptide in CFA at the tail base 24 h following the first DT injection. 7 days after immunization, inguinal LNs were collected and made into a single cell suspension. Single cell suspensions were restimulated with 50 ng/ml PMA and 500 ng/ml ionomycin in the presence of GolgiPlug for 4 h and then stained for flow cytometric analysis of IL-17A and IFNγ-producing CD45.1^+^ CD4^+^ T cells. Counting beads were used to determine absolute cell count.

### *In vitro* T Cell Differentiation

Naive CD4^+^ T cells from spleens of wild-type C57BL/6J and P2rx7^−/−^ mice were sorted by magnetic separation utilizing the mouse naive CD4^+^ T cell isolation kit and performed according to manufacturer’s instructions (Miltenyi Biotec). For cytokine-skewed Th17 differentiation, naive CD4^+^ T cells were stimulated with plate-bound 10 μg/ml anti-CD3 (Bio X Cell) and 2 μg/ml anti-CD28 (BD) in the presence of 20 ng/ml IL-6 (PeproTech), 5 ng/ml TGF-β1 (Biolegend), 20 ng/ml IL-23 (Miltenyi), and 20 ng/ml IL-1β (PeproTech) (Park et al., 2005). For Th1 differentiation, 20 ng/ml IL-12 (PeproTech) was used along with anti-CD3 and anti-CD28. Following 4 days of culture, cells were washed and restimulated with 50 ng/ml PMA and 500 ng/ml ionomycin in the presence of GolgiPlug for 4 h and IL-17 and IFNγ intracellularly stained for flow cytometric analysis. To track T cell proliferation, naive CD4^+^ T cells were labelled with 5 µM CFSE (Molecular Probes, Eugene, OR) in PBS containing 5% FBS for 5 min at RT then washed 3 times and cultured in Th1 or Th17 differentiation medium.

T cell cocultured with BMDCs were performed as previously described (Veldhoen et al., 2006), with modifications. Briefly, 2.0 × 10^4^ naive CD4^+^ T cells sorted from wild-type C57BL/6J or P2rx7^−/−^ mice and 1.0 × 10^4^ BMDCs derived from wild-type C57BL/6J were cocultured in complete RPMI-1640 supplemented with 0.2 μg/ml anti-CD3, 100 ng/ml LPS (TLRgrade, from Enzo Life Sciences, Farmingdale, NY), and 1 ng/ml TGF-β1 (Biolegend) in round-bottom 96-well plates. Four days following coculture, cells were restimulated with 50 ng/ml PMA and 500 ng/ml ionomycin in the presence of GolgiPlug for 4h and stained for flow cytometric analysis of IL-17A and IFNγ-producing CD4^+^ T cells.

### Statistics

All results were analyzed with GraphPad Prism7 Software (San Diego, CA). Statistical differences were obtained by using Student’s t test for comparison of two groups and a one-way or two-way ANOVA for multiple groups. *p < 0.05, **p < 0.01, ***p < 0.001, ****p < 0.0001.

## Results

### Global Blockade of P2X7R Does Not Compromise Antigen-specific Th17 Responses

**FIGURE 1:**
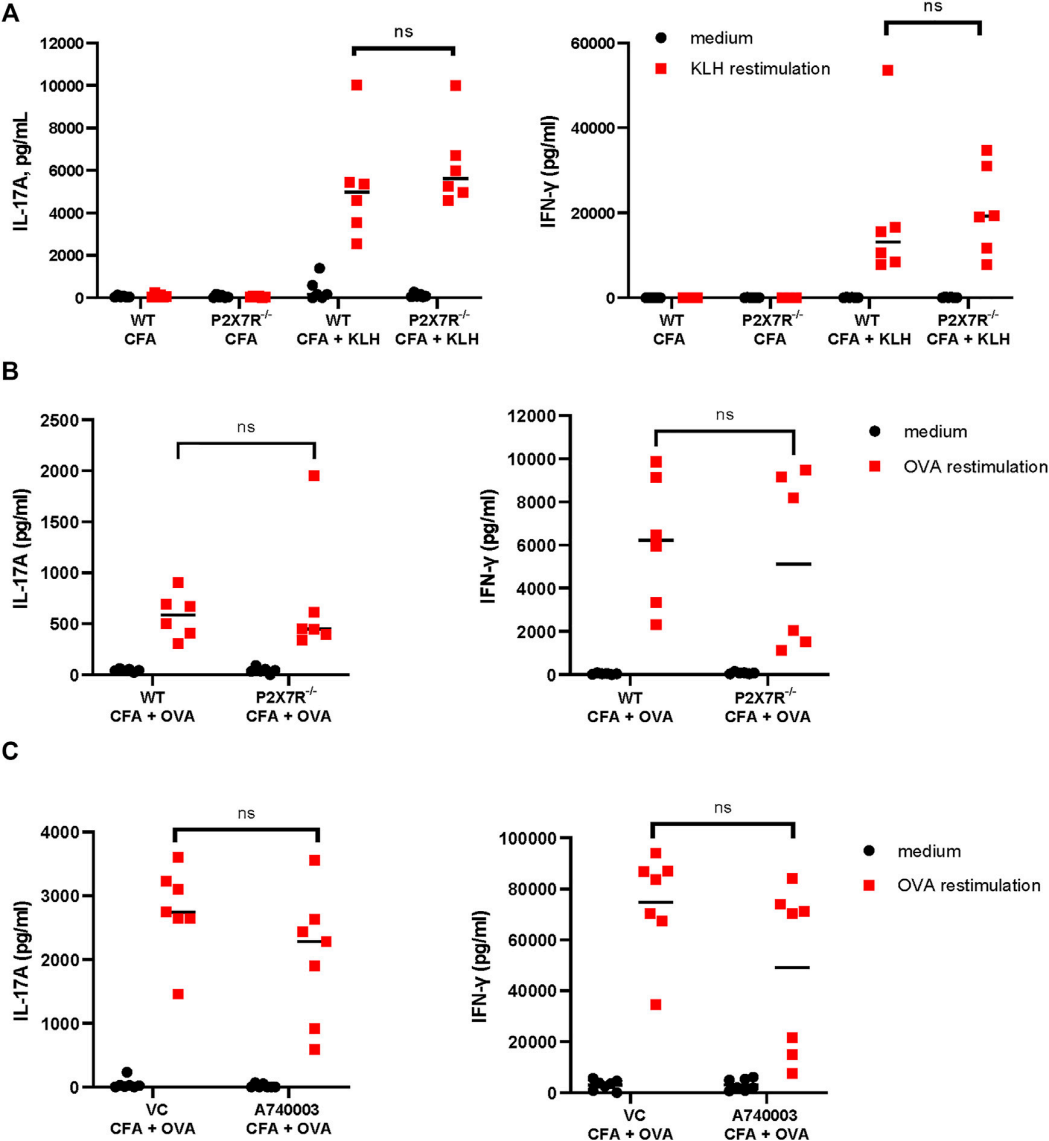
P2rx7^−/−^ and WT mice mount comparable Th17 responses. **(A)** WT C57BL/6 mice and P2rx7^−/−^ mice were immunized with 20 µg of keyhole limpet hemocyanin (KLH) emulsified in complete Freund’s adjuvant (CFA). Control mice were treated with CFA only. On day 7 inguinal lymph node (LN) cells were collected and restimulated with KLH. On day 3 supernatants were assessed for IL-17A and IFN-γ by ELISA. Results shown are a combination of two independent experiments, with each dot representing an individual mouse. **(B)** C57BL/6 mice and P2rx7^−/−^ mice were immunized with 20 µg OVA_323-339_ peptide emulsified in CFA. OVA-specific IL-17A and IFN-γ responses were analyzed as in **(A)**. Results shown are a combination of two independent experiments, each dot represents an individual mouse. **(C)** C57BL/6 mice were immunized with OVA_323-339_ peptide emulsified in CFA, in some groups mice were treated with P2X7R antagonist, A740003. LNs were collected and restimulated with OVA_323-339_ peptide as in **(A)**. Each dot represents an individual mouse from one experiment, LN cells from each mouse were restimulated in 2–7 separate wells and then averaged. NS, not significant.

P2X7R has been implicated in many inflammatory diseases, however, there is still a limited understanding of the role of P2X7R in Th cell differentiation, particularly, Th17 differentiation. To evaluate the contribution of P2X7R to Th17 immune response, we compared the IL-17A response to keyhole limpet hemocyanin (KLH) in WT mice and P2rx7^−/−^ mice. KLH has been demonstrated to induce substantial Th17 responses (Park et al., 2005; Sutton and Kemp, 2006). Thus, mice were immunized with KLH/CFA for 7 days and then IL-17A and IFN-γ production were measured in antigen-specific recall responses. The results demonstrate that T cells from P2rx7^−/−^ mice are capable of mounting an antigen-specific IL-17 response comparable to that of WT mice (Figure 1A). There was also no difference in IFN-γ production from the T cells from the two mouse strains. To determine if this response is generalized to other antigens, a similar experiment was conducted using OVA_323-339_ peptide to immunize mice (Figure 1B). Consistently, P2rx7^−/−^ mice and WT mice produced comparable levels of IL-17A and IFN-γ. However, studies have previously demonstrated a dichotomy between P2rx7^−/−^ mice and chemical P2X7R inhibitors (De Marchi et al., 2019). To confirm our findings in P2rx7^−/−^ mice we utilized a P2X7R antagonist, A740003, in the CFA + OVA model. Similar to P2rx7^−/−^ mice (Figure 1B), intraperitoneal injections of A740003 did not affect either IL-17A or IFN-γ following *in vitro* restimulation with OVA_323-339_ peptide, compared to vehicle controls (Figure 1C). To further scrutinize the ability of P2X7R to induce Th17 differentiation, we assessed the role of P2X7R expression separately on DCs and CD4^+^ T cells.

### *In vitro* Stimulation of P2X7R on DCs Enhances IL-17A Production by Allogeneic T Cells

**FIGURE 2:**
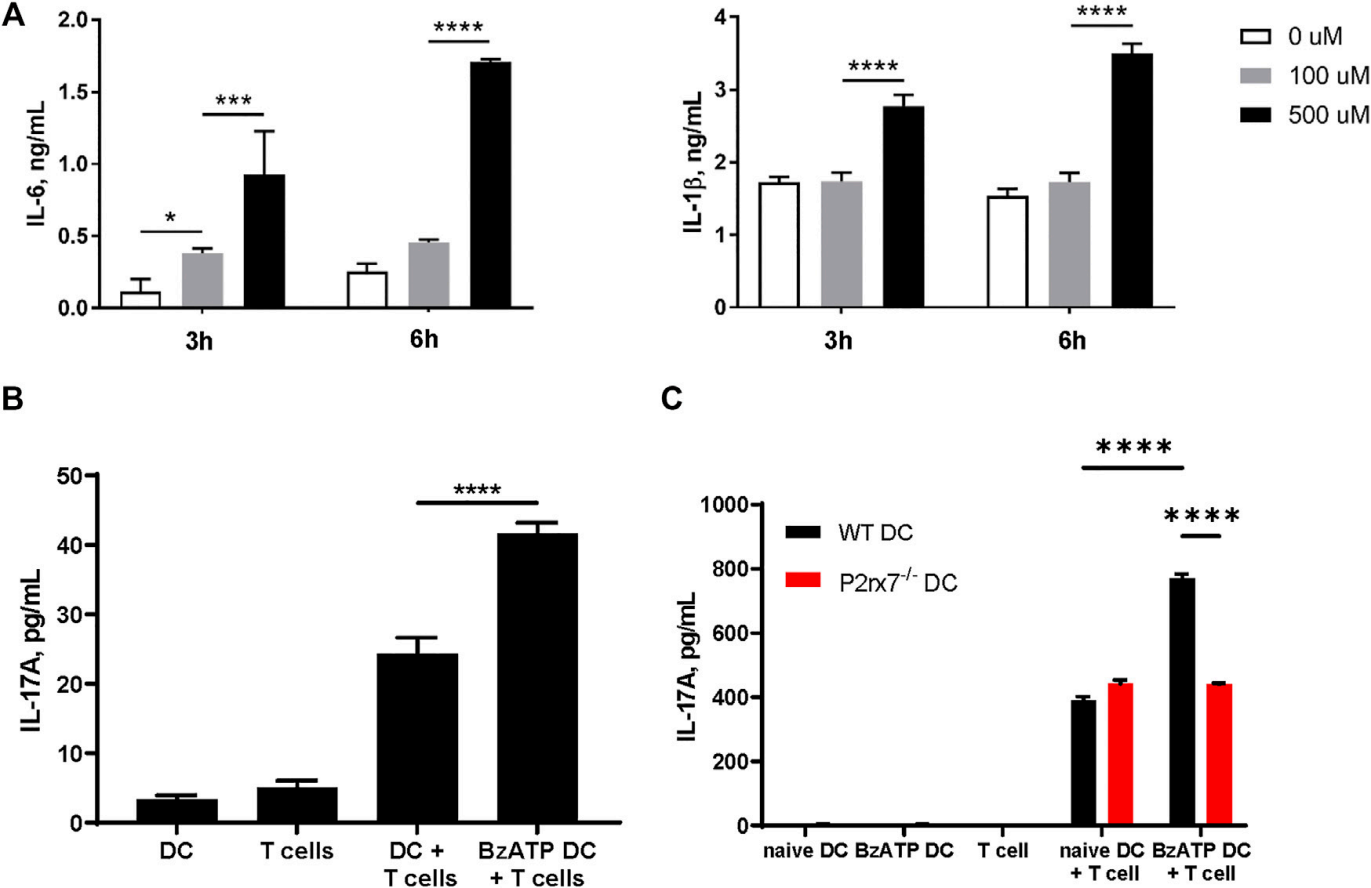
Stimulation of P2X7R on DCs can promote IL-17 production *i* *n vitro*. **(A)** Mouse BMDCs derived from C57BL/6J mice were stimulated with the indicated doses of BzATP and supernatants were collected 3 and 6 h later to determine IL-6 and IL-1β production by ELISA. **(B)** Mixed leucocyte reactions (MLR) were induced by co-culturing naive CD4^+^ T cells isolated from BALB/cJ and BMDCs derived from C57BL/6J mice, pretreated with BzATP (300 µM) or left untreated (controls) for 4 days. Supernatants were collected and L-17A levels assessed by ELISA. **(C)** BMDCs derived from WT or P2rx7^−/−^ mice, pretreated with BzATP or left untreated (controls), were cocultured with naive CD4^+^ T cells from BALB/cJ for 4 days. Supernatants were collected to assess IL-17A levels by ELISA. Results expressed as the mean ± SEM from one representative of three independent experiments. For each experiment isolated cells from several mice were pooled, co-cultures were run in triplicate wells, and the ELISA was performed in duplicates for each well. *p < 0.05, ***p < 0.001, ****p < 0.0001; NS, not significant.

Our previous work has shown that human skin migratory DCs (smiDCs) respond to P2X7R stimulation by producing proinflammatory cytokines and inducing the secretion of IL-17A from allogeneic T cells (Killeen et al., 2013). To determine if mouse DCs have a similar response, we stimulated bone marrow-derived DCs (BMDCs) from WT mice with varying doses of BzATP, an ATP analog and P2X7R agonist, and collected cell culture supernatants at the indicated time-points to examine the production of IL-6 and IL-1β, which are important for Th17 differentiation. In accordance with our previous human data, BzATP significantly induced both IL-6 and IL-1β production by mouse BMDCs in a dose dependent manner (Figure 2A). In addition, a mixed leukocyte reaction (MLR) was performed to determine if mouse BMDCs stimulated with BzATP had the capacity to induce allogeneic Th17 differentiation. The results indicate that DCs pretreated with BzATP significantly increased the production of IL-17A, compared to BMDCs not pretreated with BzATP (Figure 2B). To demonstrate that BzATP-enhanced IL-17A production is dependent on DC expression of P2X7R, we expanded BMDCs derived from WT and P2rx7^−/−^ mice and performed a similar MLR. BzATP treatment of WT BMDCs significantly increased the production of IL-17A, whereas BzATP had no effect on P2rx7^−/−^ BMDCs (Figure 2C). In the absence of exogenous BzATP, there was no significant difference between WT BMDCs and P2rx7^−/−^ BMDCs in IL-17A production, indicating that endogenous ATP is not contributing to the Th17 response (Figure 2C). Collectively, these data indicate that BzATP stimulation of DCs can enhance IL-17A production of allogeneic T cells in a P2X7R dependent manner.

### *In vivo* P2X7R Expression on Classical DCs Is Dispensable for Th17 Development

**FIGURE 3:**
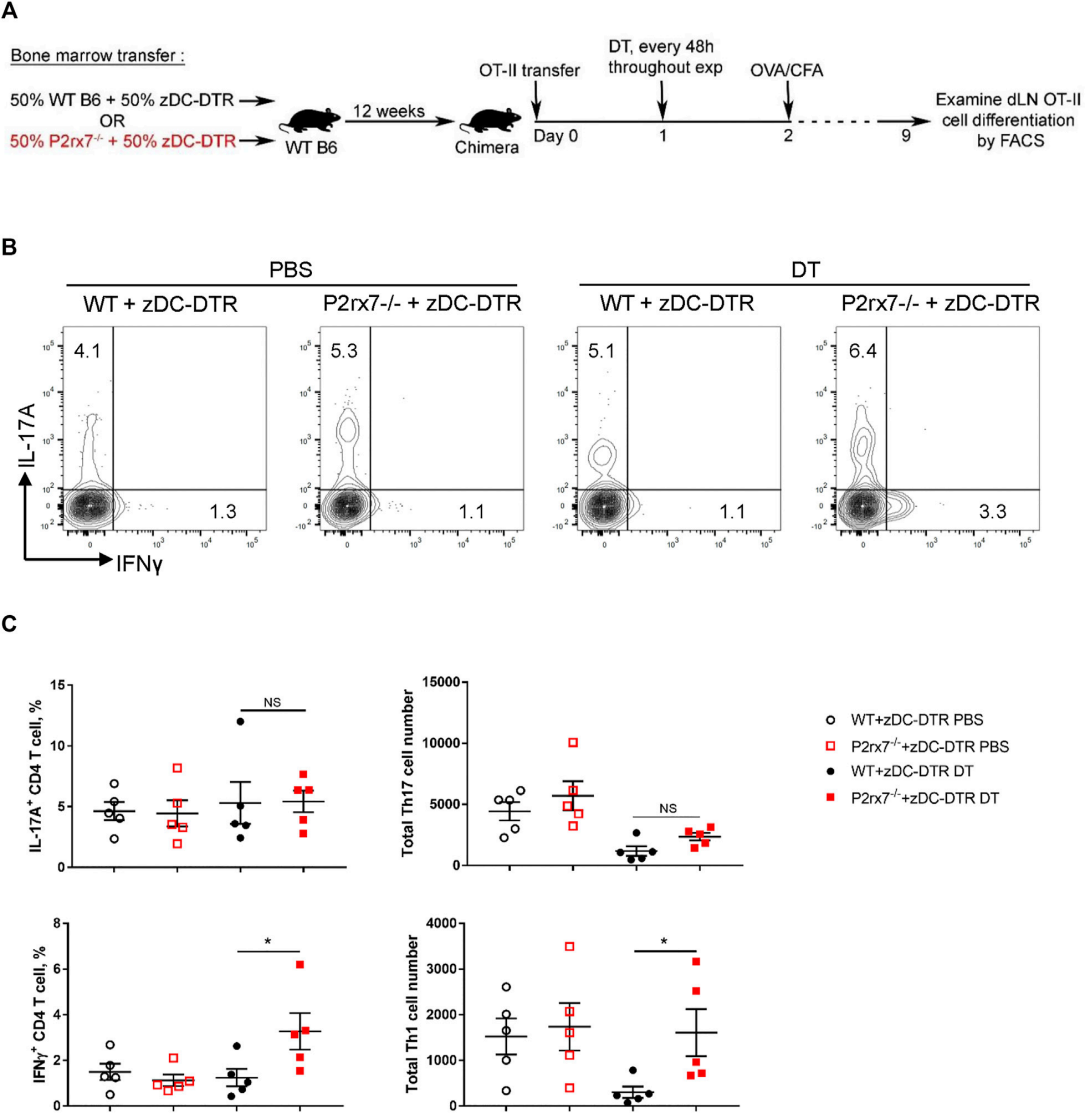
P2X7R on cDCs is not required *in vivo* for Th17 differentiation. **(A–C)** Mixed bone marrow chimeras were established by reconstituting irradiated WT mice with bone marrow from either WT or P2rx7^−/−^ mice and zDC-DTR mice mixed at 1:1 ratio (WT + zDC-DTR and P2rx7^−/−^ + zDC-DTR, respectively). After 10 weeks, chimeras were intravenously transferred with 1 × 10^5^ naive OT-II CD4^+^ T cells 1 day prior to injection with diphtheria toxin (DT) or vehicle. DT was injected every 48 h throughout the course of the experiment. 24 h following initial DT treatment, chimeras were immunized with OVA_323-339_/CFA, according to schematic **(A)** On day 9 inguinal LNs were collected, restimulated, and analyzed by flow cytometry. **(B)** Representative flow contour plots. Cells were gated on CD3^+^ CD4^+^ CD45.2^+^ CD45.1^+^ OT-II cells. **(C)** The percentage of IL-17A^+^ and IFNγ^+^ OT-II T cells and cell number were quantitated. Results shown are mean ± SEM, each dot represents an individual mouse. One representative of two independent experiments. *p < 0.05, **p < 0.01; NS, not significant.

To assess the requirement of DC-expressed P2X7R for Th17 differentiation *in vivo*, we created an *in vivo* system in which P2X7R deficiency is restricted to only classical DCs (cDCs), utilizing the zDC-DTR mice (Meredith et al., 2012). Zbtb46 (zDC) is a zinc finger transcription factor that is confined to the cDC lineage. Thus, diphtheria toxin (DT) treatment specifically ablates both CD8^+^ cDCs (CD103^+^ cDCs in nonlymphoid tissues) and CD11b^+^ cDCs subsets, while sparing monocyte-derived DCs, plasmacytoid DCs, macrophages, Langerhans cells, and NK cells. Utilizing the zDC-DTR mice we generated two groups of mixed bone marrow chimeras (Figure 3A). Specifically, we reconstituted irradiated wild type C57BL/6J mice with bone marrow cells from WT mice and zDC-DTR mice (WT + zDC-DTR; 1:1 ratio) or from P2rx7^−/−^ mice and zDC-DTR mice (P2rx7^−/−^ + zDC-DTR; 1:1 ratio). Following successful reconstitution chimeras were treated with DT to deplete cDCs (Supplementary Figure S1); thus, the cDC populations remaining are P2X7R deficient (cDC-specific P2X7R deficiency). We then utilized these chimeras to evaluate OVA-specific T cell responses in mice immunized with CFA + OVA (Figure 3A). Following re-stimulation, DT-treated P2rx7^−/−^ + zDC-DTR chimeras produced a comparable percentage and absolute number of IL-17A^+^ OT-II cells as those recovered from DT-treated WT + zDC-DTR chimeras (Figures 3B,C). Whereas P2rx7^−/−^ + zDC-DTR chimeras induced a significantly higher percentage and absolute number of IFNγ^+^ OT-II cells compared to WT + zDC-DTR chimeras. Taken together, these *in vivo* results indicate that P2X7R expression on cDCs is not required for Th17 differentiation but P2X7R signaling on cDCs does block Th1 cell development.

### P2X7R on T Cells Is Necessary for Th17 Development *in vitro*

**FIGURE 4:**
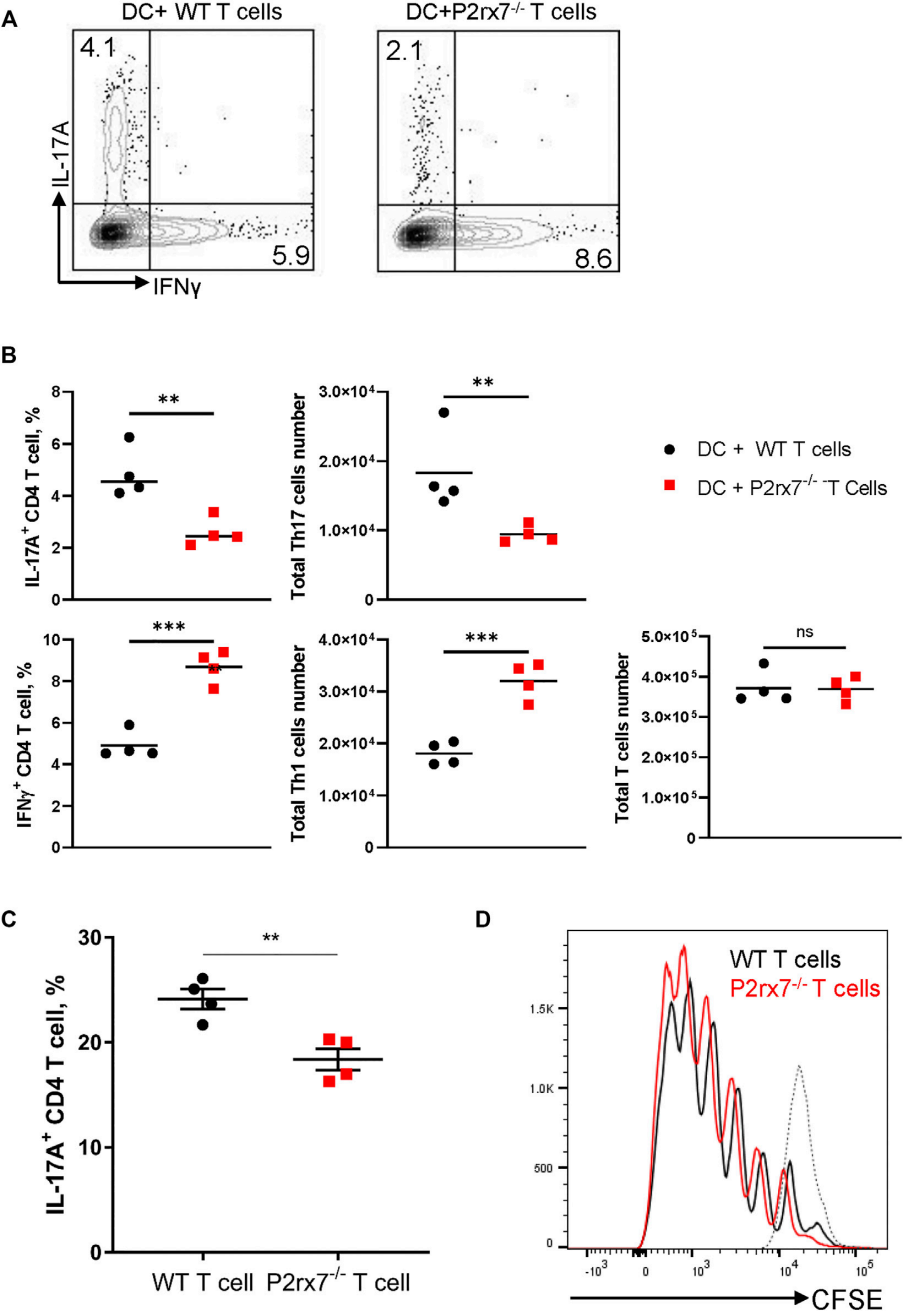
Stimulation of P2X7R on CD4^+^ T cells enhances Th17 development *in vitro*. BMDCs and naive CD4^+^ T cells from either WT or P2rx7^−/−^ mice were cocultured in the presence of 0.2 μg/ml anti-CD3, 100 ng/ml LPS, and 1 ng/ml TGF-β1 for 4 days. Cells were harvested, restimulated, and stained. IL-17A^+^ and IFNγ+CD4^+^T cell percentages were analyzed by flow cytometry **(A)** Representative flow cytometric plots gated on CD3^+^ CD4^+^ T cells. **(B)** Percentage and total cell number of IL-17A^+^ and IFNγ^+^ CD4^+^T cells were quantitated. **(C)** Naive CD4^+^ T cells isolated from WT or P2rx7^−/−^ mice were stimulated with plate-bound anti-CD3 (10 μg/ml) and anti-CD28 (2 μg/ml) in the presence of Th17-skewing cytokines for 4 days. Percentages of IL-17A^+^ T cells were determined by flow cytometry following restimulation. **(D)** Naive CD4^+^ T cells isolated from WT or P2rx7^−/−^ mice, pre-labelled with CFSE, were stimulated as in **(C)** for 4 days. T cell proliferation was analyzed by CFSE intensity in WT and P2rx7^−/−^ T cells. Dotted line represents CFSE labeled but unstimulated naive T cell. Results shown as mean ± SEM. **(A,B)** are one representative of five independent experiments **(C,D)** are one representative of three independent experiments. *p < 0.05, **p < 0.01, ***p < 0.001; NS, not significant.

It has been reported that P2X7R is involved in T cell activation through regulation of the autocrine ATP pathway (Yip et al., 2009) and it is possible that the P2X7R on T cells itself directly participates in Th17 differentiation. To test this hypothesis, we utilized a Th17 differentiation coculture system (Veldhoen et al., 2006). Naive WT or P2rx7^−/−^ CD4^+^ T cells were co-cultured with WT BMDCs in the presence of anti-CD3ε, TGFβ, and LPS. P2rx7^−/−^ T cells demonstrated significantly impaired Th17 development but enhanced Th1 responses in terms of both the positive cell percentage and total cell number of IL-17^+^ and IFNγ^+^ cells (Figures 4A,B). To confirm this conclusion, we next performed a cytokine skewed Th17 differentiation assay to test the requirement of P2X7R on CD4^+^ T cells. We purified naive CD4^+^ T cells from either WT or P2rx7^−/−^ mice and stimulated them with anti-CD3 plus anti-CD28 in the presence of Th17-skewing cytokines IL-6, TGFβ, IL-1β, and IL-23. The percentage of IL-17A-producing T cells was significantly reduced in absence of P2X7R when compared with controls (Figure 4C), is spite of equivalent proliferation (Figure 4D). Thus, together these *in vitro* results indicate that P2X7R expression on T cells is necessary for Th17 differentiation while inhibiting Th1 development.

### *In vivo* Th17 Differentiation Is Not Increased by P2X7R Expression on T Cells

**FIGURE 5:**
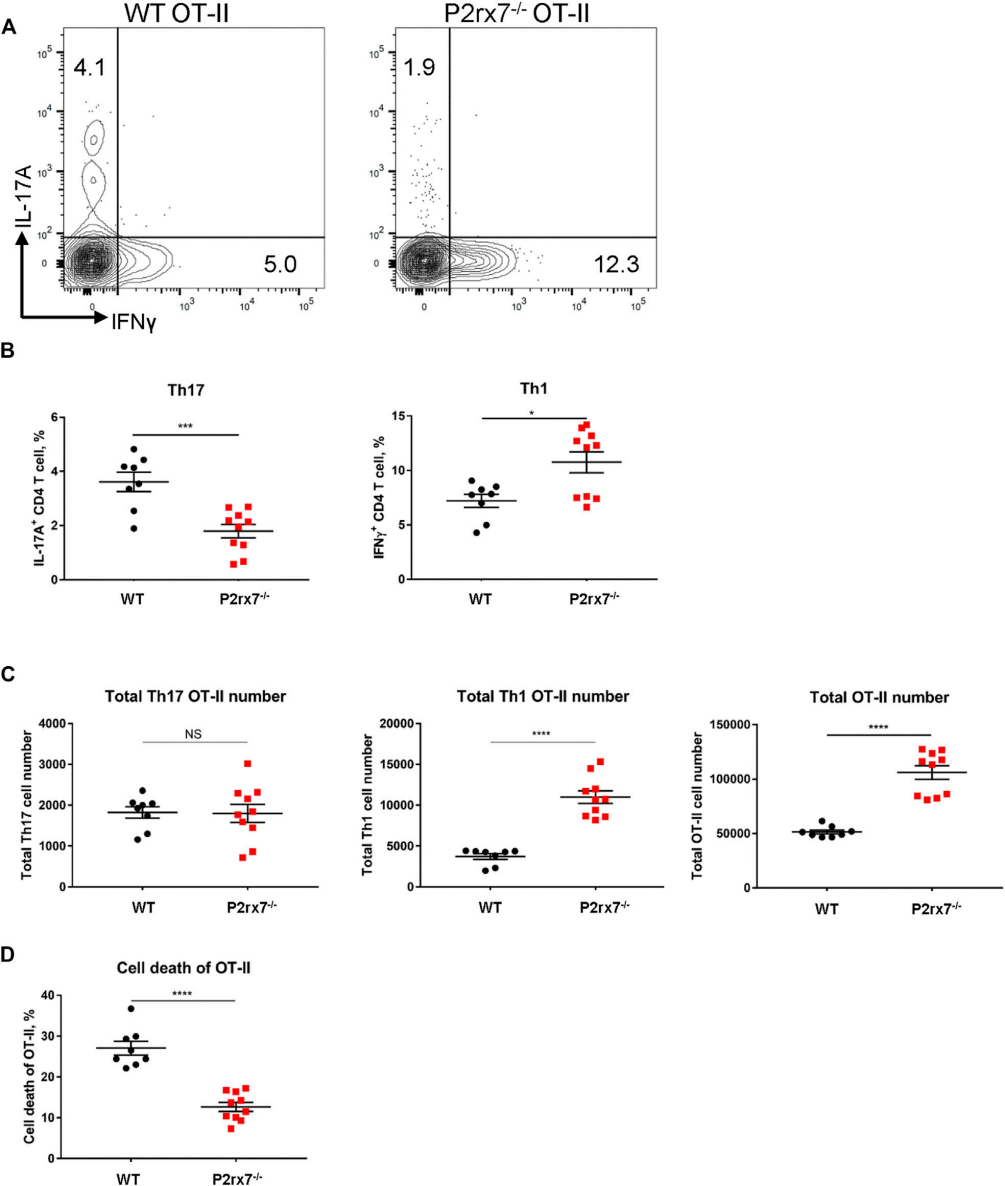
*In vivo* P2X7R expression on CD4^+^ T cells is not essential for Th17 differentiation. **(A–D)** Naive WT or P2rx7^−/−^ OT-II CD4^+^ T cells (both CD45.2) were adoptively transferred intravenously into congenic WT CD45.1 mice. The following day mice were immunized with OVA_323-339_/CFA. Inguinal LNs were collected and restimulated 7 days post-immunization. **(A)** Representative flow cytometric contour plots, cells were gated on CD3^+^ CD4^+^ CD45.2^+^ OT-II cells. **(B,C)** The percentage and total cell number of IL-17A^+^ and IFNγ^+^ OT-II T cells in the CD45.2 gate, were quantitated. **(D)** Cell death quantified by viability staining dye. Results shown as mean ± SEM, each dot represents an individual mouse. One representative of three independent experiments. *p < 0.05, ***p < 0.001, ****p < 0.0001; NS, not significant.

To address the *in vivo* physiological relevance of P2X7R expression on T cells in differentiation of Th17 cells, we generated P2rx7^−/−^ OT-II mice and examined differentiation of CD4^+^ T cells following adoptive transfer of WT and P2rx7^−/−^ OT-II T cells into congenic wild type mice and then immunizing with CFA + OVA. As demonstrated in Figures 5A,B significantly lower percentage of IL-17A^+^ T cells were detected following adoptive transfer of P2rx7^−/−^ OT-II cells compared to WT OT-II cells, whereas the percentage of IFNγ^+^ T cells was significantly higher in the P2rx7^−/−^ OT-II population. Notably, the absolute number of P2rx7^−/−^ OT-II cells was significantly higher than that of WT OT-II cells (Figure 5C). Therefore, in spite of the difference in percentage, the absolute number of Th17 cells was unchanged between groups, while the absolute number of P2rx7^−/−^ OT-II Th1 cells was significantly higher.

Because P2X7R expression has been shown to induce T cell death (Hubert et al., 2010; Hashimoto-Hill et al., 2017; Faliti et al., 2019), we hypothesized that the increase in OT-II cell number associated with a loss of P2X7R could be attributed to a decrease in cell death. To assess the cell death of OT-II cells, we utilized a viability dye. Consistent with previous reports, WT OT-II cells demonstrated a much higher percentage of cell death compared to P2rx7^−/−^ OT-II cells (Figure 5D), which lead to a larger population of P2rx7^−/−^ OT-II cells (Figure 5C). It should be noted that cell death was an *in vivo* event that was not due to isolation procedures as the only population of cells that demonstrated a significant increase in cell death were adoptively transferred WT OT-II cells compared to adoptively transferred P2rx7^−/−^ OT-II cells and host cells (Supplementary Figure S2). Thus, *in vivo* studies indicate that P2X7R expression on T cells does not enhance the Th17 cell development but does suppress Th1 differentiation, possibly through cell death as a self-limiting mechanism to constrain inflammation.

## Discussion

The purinergic P2X7R is significant in inflammasome activation and generally considered to favor proinflammatory immune responses. In this regard, P2X7R contributes to Th17 responses in murine colitis, murine arthritis, murine and human psoriatic skin inflammation, allograft rejection, and human adipose tissue inflammation (Atarashi et al., 2008; Killeen et al., 2013; Fan et al., 2016; Pandolfi et al., 2016; Diaz-Perez et al., 2018; Fan et al., 2021). However, there is still a limited understanding of the role of P2X7R in Th cell differentiation, particularly, Th17 differentiation. For instance, studies using P2rx7^−/−^ mice demonstrate that P2X7R deficiency does not alleviate inflammation but alternatively enhances inflammation. It is possible that the discrepancy is caused by the utilization of different models with different dependencies on CD4^+^ T cells, global knockout of P2X7R versus pharmacological blockade, and the expression of P2X7R on various cell populations (Cekic and Linden, 2016; De Marchi et al., 2019; Grassi and De Ponte Conti, 2021). In this regard, P2X7R is expressed on both inflammatory and anti-inflammatory cells, such as myeloid derived suppressor cells (MDSCs) and T regulatory cells (Bianchi et al., 2014; Cekic and Linden, 2016; Li et al., 2020; Grassi and De Ponte Conti, 2021). Moreover, ATP and BzATP can both be metabolized to adenosine which is generally considered an anti-inflammatory agent but can induce differential responses, for instance adenosine signaling through the A_2B_ receptor can induce inflammation (Santiago et al., 2020). Regarding P2X7R^−/-^ mice and pharmacological blockade, herein, the results demonstrate that global P2X7R signaling blockade, either through P2rx7^−/−^ mice or systemic pharmacological antagonism, leads to comparable Th17 and Th1 immune responses with that of WT mice. Consequently, systemic P2X7R signaling might not be demonstrating an inflammatory effect due to counteracting anti-inflammatory mechanisms that mask the phenotype. To further gain a better understanding of P2X7R signaling in Th17 differentiation and to assess the role of P2X7R signaling on different cellular populations we independently interrogated the effects of P2X7R expression on DCs and CD4^+^ T cells.

Perhaps the earliest connection between ATP and IL-1β proteolytic maturation and release came from a study demonstrating that ATP induced the secretion of the mature form of IL-1β from LPS-stimulated mouse peritoneal macrophages (Hogquist et al., 1991). Later studies utilizing macrophages demonstrated that ATP-induced K^+^ efflux was critical for IL-1β processing (Perregaux and Gabel, 1994). ATP’s effect was also linked to the activation of the inflammasome and caspase-1, which cleaves pro-IL-1β to produce its active form (Di Virgilio et al., 2017). It is proposed that ATP provides the second signal with the first signal being the stimulation of pro-IL-1β production through NF-κB, provided by microbial components (Franchi et al., 2010). P2X7R, as an ATP receptor, was introduced into this “Two signal model” almost at the same period (Ferrari et al., 1997; Solle et al., 2001; Franchi et al., 2007). These studies clearly demonstrate the relationship between P2X7R and IL-1β in macrophages. We have previously demonstrated a similar relationship using human smiDCs. In this study, smiDCs stimulated through the P2X7R expressed IL-1β and IL-6, which were blocked following the addition of P2X7R antagonists (Killeen et al., 2013). In the current study, BzATP stimulation of BMDCs can also induced IL-1β and IL-6 secretion. Our results herein extend the observation that ATP drives IL-1β release in dendritic cells as well as macrophages.

Following P2X7R stimulation on BMDCs, we demonstrate that DCs can significantly increase the IL-17A production and Th17 differentiation in naive CD4^+^ T cells. These findings are also in line with our previous studies utilizing human smiDCs, in which we determined that purinergic signaling provokes innate cutaneous inflammatory responses, DC17 differentiation, and Th17 responses (Killeen et al., 2013). Of note, in the absence of exogenously added BzATP, P2rx7^−/−^ BMDCs are capable of inducing comparable levels of IL-17A from co-cultured T cells with that of WT BMDCs, albeit at significantly lower levels, indicating that BMDCs likely have compensatory mechanisms for biasing Th17 responses. This notion is supported by our *in vivo* data that indicates that cDC-specific P2X7R deficiency does not affect the percentage of IL-17A^+^ T cells. Thus, combining the *in vitro* and *in vivo* findings, the data suggests that P2X7R expression on DCs is not necessary for Th17 development, but can augment Th17 development. These results do not rule out a role for radio-resistant LCs and other non-cDCs expressing P2X7R. Concordantly, our previous studies demonstrated that human LCs can induce Th17 differentiation (Mathers et al., 2009). However, the model of systemic immunization utilized in these studies is likely unaffected by cutaneous LCs. Moreover, cDCs especially CD11b^+^ cDCs, express higher levels of genes involved in antigen presentation, are found at most environmental interfaces, and are thought to be responsible for the induction of Th17 responses in many models, including the CFA-immunization models utilized in our studies (Merad et al., 2013).

In terms of a Th1 response, the *in vivo* data indicates that cDC-specific P2X7R deficiency induces a significantly higher percentage of IFNγ^+^ cells compared with P2X7R-sufficient cDCs, demonstrating that DC signaling through the P2X7R leads to the suppression of Th1 responses. These results are consistent with previous findings by la Sala et al. (2001).

To directly examine the role of P2X7R expression on T cells we utilized *in vitro* co-cultures and *in vivo* adoptive transfer of P2rx7^−/−^ OT-II T cells. Corresponding to the DC findings, P2X7R expressed by CD4^+^ T cells also promotes Th17 development *in vitro* but P2X7R expression on CD4^+^ T cells is not essential *in vivo* for Th17 differentiation. Differences between *in vitro* and *in vivo* are likely due to compensatory mechanisms or mechanisms of immune resolution, such as adenosine, MDSCs, and T regulatory cells (Bianchi et al., 2014; Cekic and Linden, 2016; Li et al., 2020; Santiago et al., 2020; Grassi and De Ponte Conti, 2021). However, we do demonstrate that Th1 responses are suppressed *in vitro* and *in vivo* by P2X7R expression on CD4^+^ T cells. Importantly, in accordance with previous reports (Seman et al., 2003; Faliti et al., 2019), we determined that P2X7R expression on T cells can induce T cell death *in vivo* seemingly resulting in the decrease of Th1 cells but not Th17 cells; findings that suggest Th1 cells are more sensitive to P2X7R-induced cell death. We hypothesize that this difference in sensitivities is due to the activation of the programed death-1 (PD-1) and PD-1 ligand (PD-L1) signaling pathway. In this regard, prior studies have demonstrated that the PD-1/PD-L1 signaling pathway leads to an increase in Th1 cell death and no effect on Th17 cells (Singh et al., 2013; Stephen-Victor et al., 2016). Overall, our study indicates that P2X7R can enhance but is not required for Th17 differentiation. These findings contribute to our understanding of P2X7R signaling and suggest cell-type specific targeting strategies could improve the clinical efficacy of P2X7R therapeutics.

## Data Availability

The raw data supporting the conclusion of this article will be made available by the authors, without undue reservation.
