# Supplementary material for: P2X7 Receptor Expression and Signaling on Dendritic Cells and CD4+ T Cells is Not Required but Can Enhance Th17 Differentiation
Source: Front Cell Dev Biol. 2022 Mar 8;10:687659. doi: 10.3389/fcell.2022.687659 (PMC8957928; doi:10.3389/fcell.2022.687659)
Supplement: Supplementary file 1 [file DataSheet1.PDF]

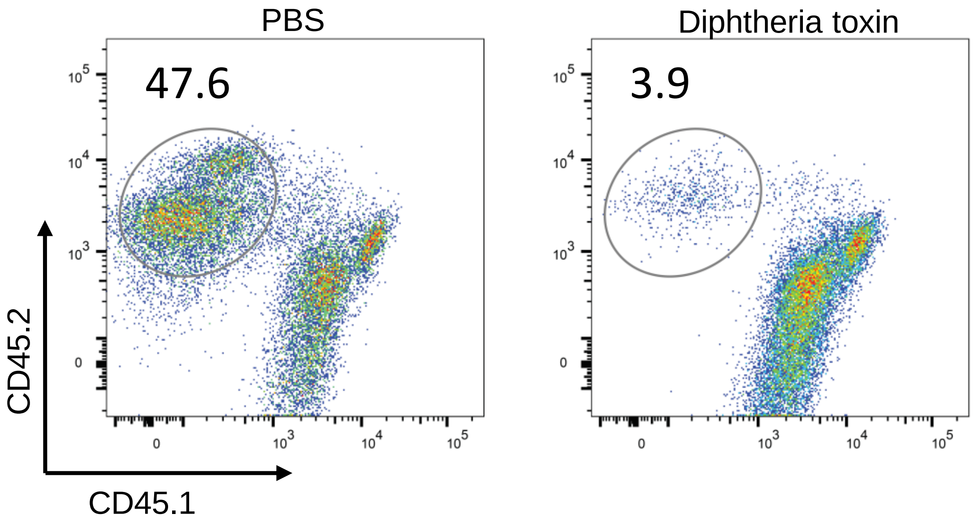

Supplementary Figure S1. Successful reconstitution of mixed bone marrow and DT depletion of classical DCs only from zDC-DTR bone marrow (CD45.2) was shown for the mixed bone marrow chimeras experiment in Fig 3 B-C. Percentage of cDC (gated on Lin^-/$CD11C^+$/MHC II^{high}) (Meredith et al., 2012) derived from CD45.2 zDC-DTR bone marrow cells is shown.

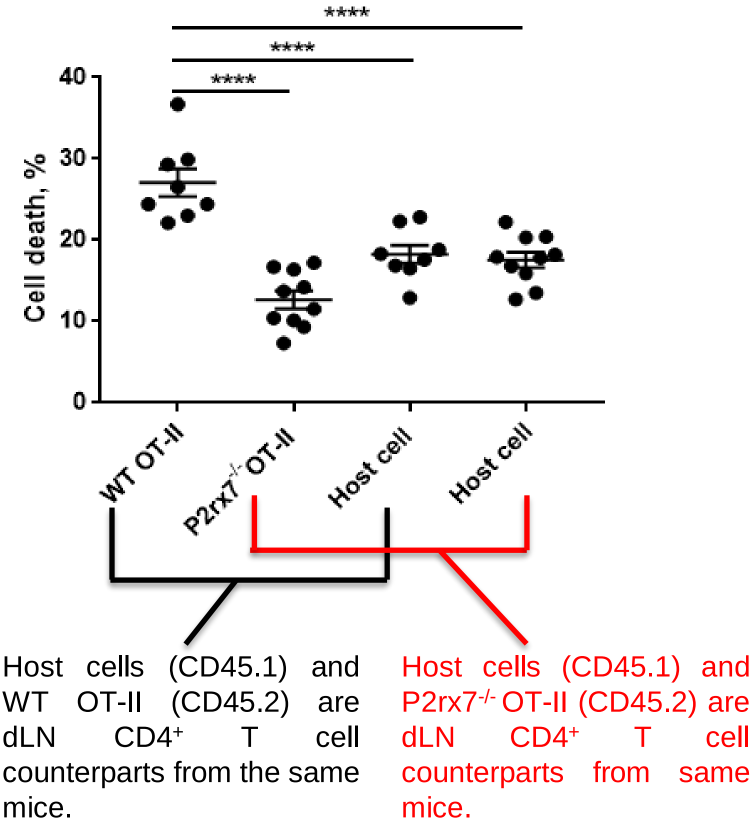

Supplementary Figure S2. Cell death is primarily an *in vivo* event during OVA-specific immune activation affecting P2X7R WT OT-II cells. Naive WT or P2rx7$^{-/-}$ OT-II CD4^{+} T cells (both CD45.2) were adoptively transferred intravenously into congenic WT CD45.1 mice. The following day mice were immunized with OVA$_{323-339}$/CFA. Inguinal LNs were collected and restimulated 7 days post-immunization. Cell death quantified by viability staining dye. Host cells (P2X7R WT) compared to adoptively transferred P2X7R WT OT-II and P2rx7$^{-/-}$ OT-II cells. Only transferred WT OT-II cells show a significant increase in cell death while host cells do not, though both cells express P2X7R. Results shown as mean ± SEM, each dot represents an individual mouse. ****$p<0.0001$.
